# A Case of Sjögren's Syndrome Mimicking Inflammatory Myopathy

**DOI:** 10.7759/cureus.3393

**Published:** 2018-10-01

**Authors:** Shaweta Khosa, Dominic A Hovsepian, Gurveer S Khosa, Yim Catherine, Bhavesh Trikamji, Shri K Mishra

**Affiliations:** 1 Neurology, Olive View - University of California Los Angeles Medical Center, Los Angeles, USA; 2 Neurology, David Geffen School of Medicine at University of California, Los Angeles, USA; 3 Internal Medicine, Indira Gandhi Medical College, Shimla, IND; 4 Radiology, Olive View - University of California Los Angeles Medical Center, Sylmar, USA; 5 Neurology, Harbor– University of California Los Angeles Medical Center, Torrance , USA; 6 Neurology, Keck School of Medicine of the University of Southern California, Los Angeles, USA

**Keywords:** myopathy, skeletal muscle, neuropathy, muscle biopsy, sjögren's syndrome

## Abstract

Sjögren's syndrome (SS) is a chronic autoimmune disorder, characterized by lymphocytic infiltration of exocrine glands and causing the decreased function of lacrimal and salivary glands. We describe a case of a 34-year-old male who presented with Sjögren's syndrome presenting as myopathy and sensorimotor neuropathy. His creatinine kinase levels were elevated with positive anti-Sjögren's syndrome-related antigen A autoantibodies and anti-Sjögren's syndrome Type B autoantibodies. Electromyography showed evidence of irritable myopathy. Parotid gland biopsy demonstrated focal lymphocytic sialadenitis. The patient favorably responded to high-dose steroids. Thus, although rare, inflammatory myopathy must be considered part of the initial presentation of Sjögren's syndrome.

## Introduction

Primary Sjögren's syndrome (SS) is a chronic autoimmune disorder with lymphocytic infiltration of the exocrine glands. It usually presents as dryness of the conjunctiva and cornea (keratoconjunctivitis sicca) and dry mouth (xerostomia), fatigue, and joint pain. Neurological, musculoskeletal, pulmonary, and gastrointestinal symptoms are seen in almost 30 - 40% of patients with primary SS [1]. Primary SS usually affects women more than men with a predominance of 9:1 and usually manifests in the fifth decade of life [2]. SS can be found in association with other systemic autoimmune diseases, such as systemic lupus erythematosus (SLE), scleroderma, rheumatoid arthritis, or dermatomyositis that is referred to as secondary SS.

Although muscle pain and/or muscular weakness has been reported frequently in patients with primary SS, myositis has been reported in less than 3% of patients. We describe a case of primary SS that presented as an inflammatory myopathy and responded to immunosuppressive therapy. Our case highlights the fact that although myositis is rare in primary SS, this syndrome should be considered upon initial evaluation of patients presenting with unexplained myositis.

## Case presentation

A 34-year-old male presented with chief complaints of a two-month history of right-sided facial numbness, along with bilateral hand and foot numbness. He also reported xerostomia, as well as bilateral parotid gland swelling and dysphagia over the same period of time. The pain and numbness involved his feet and hands bilaterally and had been progressively worsening. Physical examination revealed normal muscle bulk and tone in all four extremities. However, distal weakness was observed with weak bilateral hand grip and he was unable to make a fist due to pain. He also had decreased sensation to light touch and pinprick in the right mandibular distribution of the trigeminal nerve. Decreased sensation to light touch, pinprick, and vibration was observed in bilateral hands (involving the second, third, and fourth digits), along with the medial and lateral forearms extending up to the elbows. The initial laboratory examination was significant for creatine kinase (CK) levels of 3,288 IU/L, erythrocyte sedimentation rate (ESR) of 60 mm/hr, C-reactive protein (CRP) of 21.2 mg/dl, and an aldolase of 17.1 IU/L. Hepatic function tests revealed an alanine aminotransferase of 233 U/L and an aspartate aminotransferase of 160 U/L. Immunological studies showed positive titers of anti-Sjögren's syndrome-related antigen A (SS-A) antibodies and anti-Sjögren's syndrome Type B (SS-B) antibodies > 8, while anti-Jo-1, anti-signal recognition particle (anti-SRP), and anti-melanoma differentiation-associated protein 5 (anti-MDA5) antibodies were negative.

**Figure 1:**
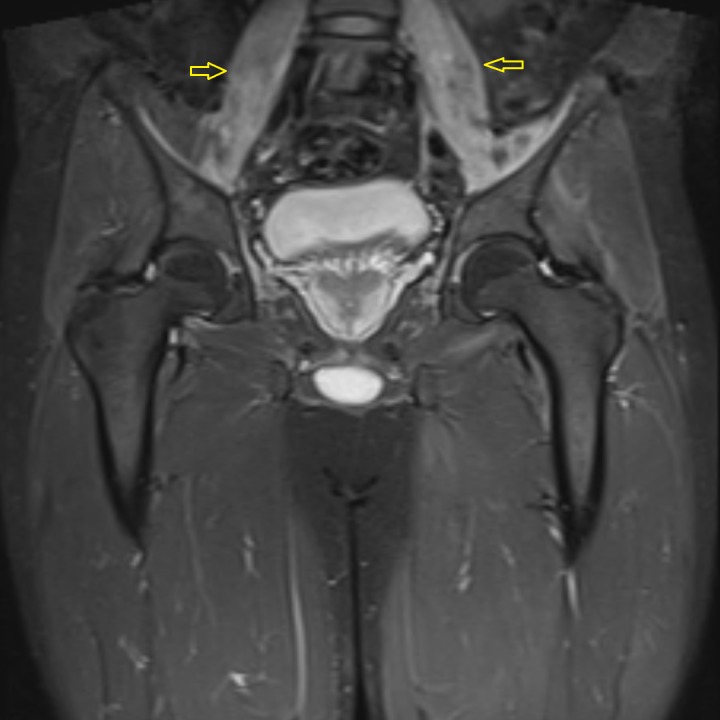
MRI pelvis (coronal view) Coronal fat-saturated, fluid-sensitive sequence without contrast showing hyperintensity in the bilateral psoas and iliacus muscles (arrows) suggestive of myositis.

**Figure 2:**
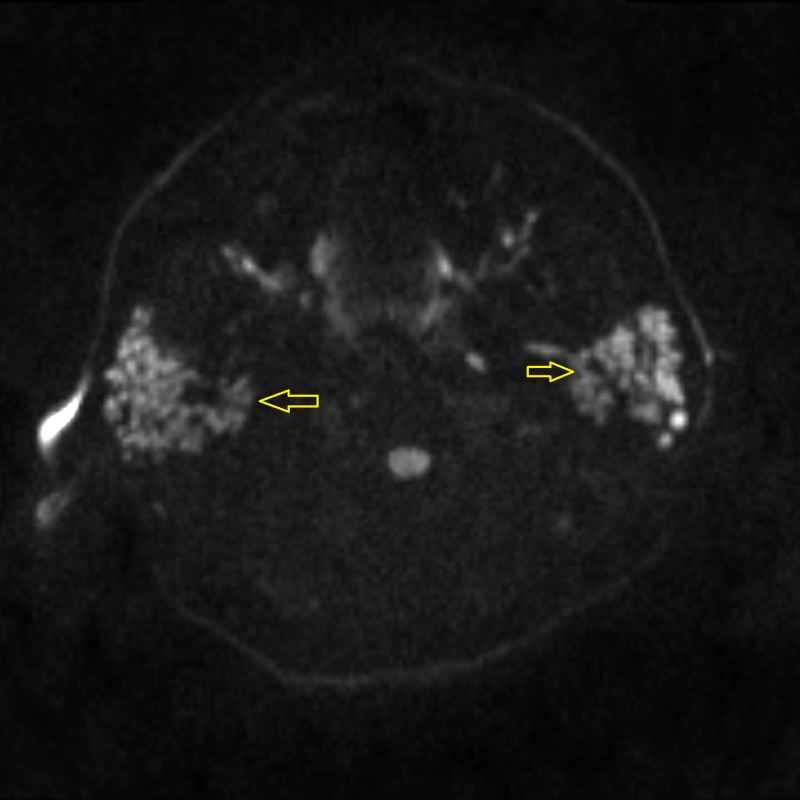
Diffusion-weighted (DW) MR imaging of the parotid gland showing hyperintense nodules in the bilateral parotid glands (arrows) suggestive of parotitis MR: magnetic resonance

**Figure 3:**
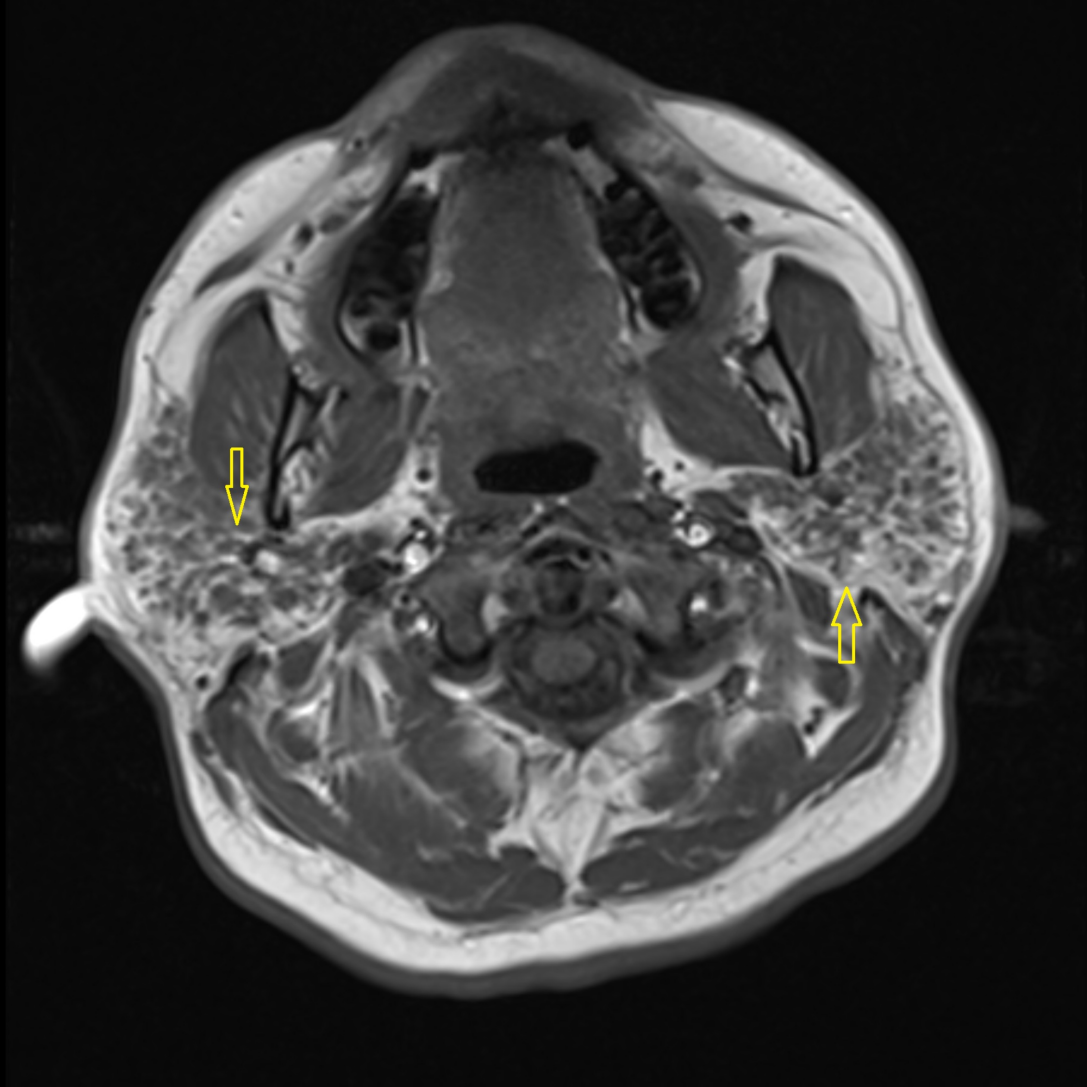
T-1 weighted image at the level of the parotid gland showing hypointense nodules in the bilateral parotid glands (arrows) suggestive of parotitis

**Figure 4:**
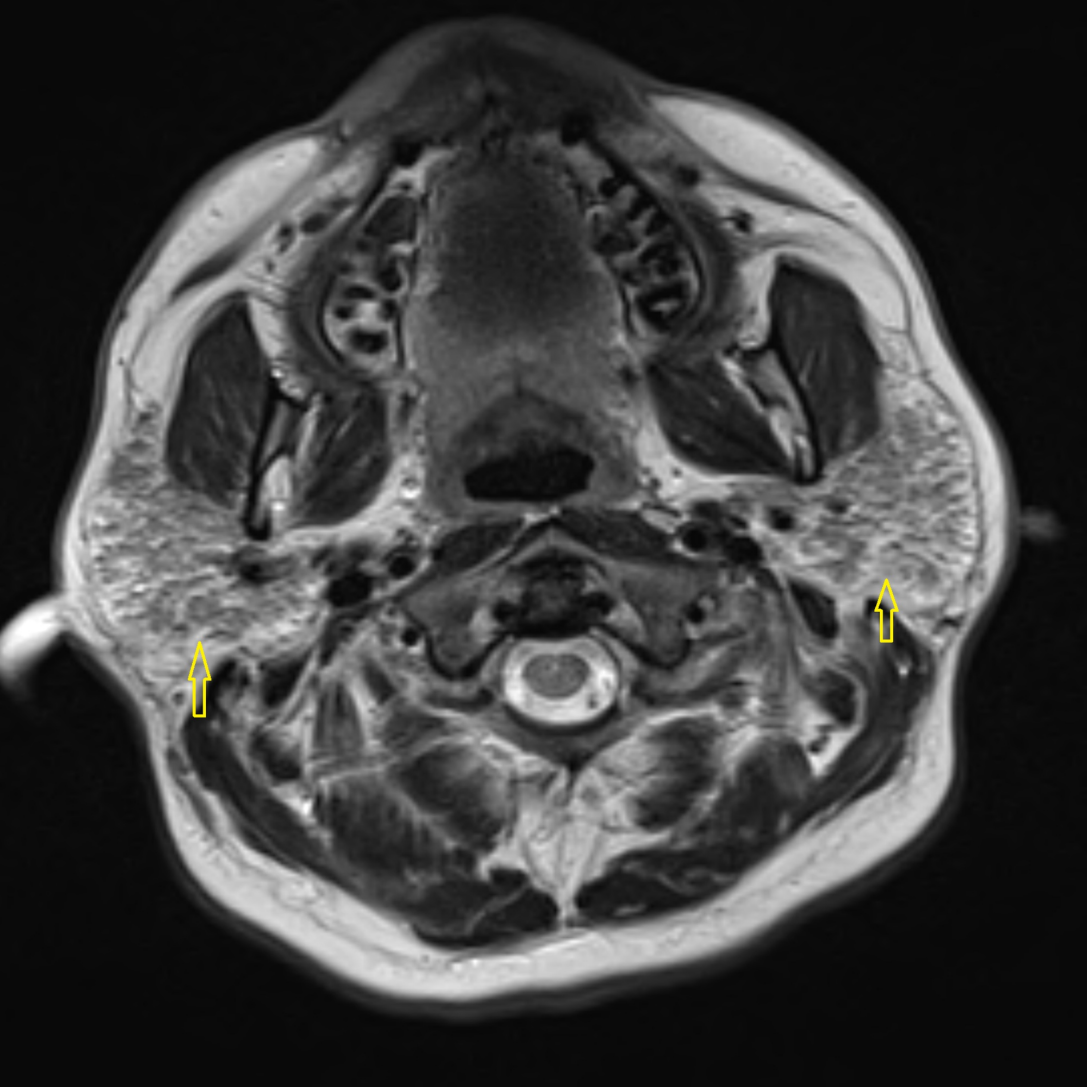
T-2 weighted image of the parotid gland showing hypointense nodules (arrows) in the bilateral parotid glands suggestive of parotitis

Needle electromyography (EMG) was performed in both upper and both lower extremities. Various muscles tested included the deltoid, biceps brachiis, triceps brachii, pronator teres, and abductor pollicis brevis in both upper extremities. In the lower extremities, the vastus lateralis, tibialis anterior, and gastrocnemius muscle groups were tested. The compound motor action potential (CMAP) was also measured in both upper and lower extremities. The EMG studies showed subclinical irritable myopathy affecting the proximal muscles in the right upper extremity more than the right lower extremity. The patient also underwent nerve conduction studies, including testing of sensory nerve action potential (SNAP) amplitude in both upper extremities and lower extremities involving median, ulnar, and sural nerves. Bilateral median nerve sensory neuropathy was observed on nerve conduction studies. A computed tomography (CT) scan of the head and neck was performed which showed mild fatty infiltration of bilateral parotid glands, along with reactive lymph nodes scattered in the neck. Magnetic resonance imaging (MRI) of the pelvis demonstrated bilateral edema of the psoas and iliacus muscles as evident in Figure 1. An MRI of the brain showed the involvement of the parotid gland, suggestive of SS, as seen in Figures 2-4. The creatinine kinase levels remained persistently high despite hydration peaking to a level of 7,215 IU/L.

Given the constellation of findings, including irritable myopathy on electromyography (EMG), positive SS-A/SS-B antibodies, and persistently elevated CK, as well with the MRI findings, a diagnosis of Sjögren's disease myositis was made. The patient was given 125 mg of intravenous methylprednisolone twice daily for three days with an improvement of his pain and numbness. The CK also trended down to 2,600 IU/L over the same period. He was subsequently switched to oral prednisone, 60 mg once daily by mouth, on discharge with a plan to taper by 20 mg every week.

The patient eventually underwent a salivary gland biopsy demonstrating focal lymphocytic sialadenitis consistent with SS. A follow-up muscle biopsy of the left deltoid showed mild changes, including occasional degenerating fibers and some denervation with no inflammatory changes. CK levels continued to remain elevated after discharge, and the patient was started on methotrexate and intravenous immunoglobulin (IVIG), in addition to oral prednisone, 20 mg. The IVIG dose was increased from 80 mg IV monthly to 150 mg IV monthly and mycophenolate mofetil, 1,500 mg twice daily, was added after his CK levels failed to normalize, despite the above treatment. The patient responded appropriately with the most recent CK level being 34 IU/L. He is currently asymptomatic and continues to follow-up with rheumatology 11 months after his initial presentation.

## Discussion

Primary SS is an autoimmune disorder classically linked to symptoms involving the exocrine glands, including dry mouth and dry eyes. Lymphocytic infiltration and immune-mediated reactions can lead to various extra-glandular manifestations in primary SS, including interstitial nephritis, interstitial pneumonitis, peripheral neuropathy, palpable purpura, arthralgias, and myalgias. Muscle involvement in secondary SS can be seen when it is associated with other autoimmune disorders, such as polymyositis, dermatomyositis, and inclusion body myositis. However, muscle involvement in primary SS has been reported to be relatively infrequent, occurring in 1 - 10% of the patients. Among this small group of primary SS patients, the prevalence of myositis has been reported being even lower. Even if myositis is present, it typically manifests as subclinical myositis [3-5].

In a recent multi-center Italian cohort study to describe the prevalence of myositis in primary SS, only six cases of myositis were histologically confirmed out of 1,320 patients with primary SS [6]. This prevalence was even less than the previously reported number of 3% of primary SS patients having myositis that was described by Kraus et al. in 1994 [3]. In another study, Lindvall et al. evaluated 48 patients with primary SS with a complaint of muscle pain in 44% of this cohort. Of these 48 patients, 36 underwent further neuromuscular evaluation, including muscle biopsies. Although 29 patients had abnormal muscle biopsies, the muscle symptoms were not related to the histological signs of myositis, suggesting that subclinical myositis is more common in primary SS [4].

Our patient presented with clinical features of SS, as well as with myalgias with elevated CK levels. The presence of myalgia and other musculoskeletal symptoms, along with SS, is usually seen in secondary Sjögren's syndrome as well as mixed connective tissue disorder (MCTD). One of the key features in suspecting MCTD is the presence of unexplained Raynaud’s phenomenon, which was absent in our patient. Physical examination was also unremarkable for other features of systemic sclerosis and systemic lupus erythematosus (SLE) which tend to be seen in MCTD. In previous case reports, myositis in SS has also been linked to polymyositis [5, 7-8]. In our patient, the myositis panel was negative, except for Ku antibodies that are usually associated with scleroderma myositis and interstitial lung disease which this patient did not have. Muscle biopsy is an important diagnostic tool to differentiate various inflammatory myopathies seen in SS and MCTD, such as polymyositis, dermatomyositis, and inclusion body myositis (IBM). The muscle biopsy in patients with primary SS can show subclinical myositis. However, the biopsy in our patient was not impressive, showing minimal activity affecting the muscle with no significant inflammation, suggesting an underlying autoimmune process. The MRI of the parotid glands showed parotitis due to Sjögren's syndrome later confirmed with a positive biopsy of the salivary glands. These findings point towards a picture of primary SS with myositis rather than the presence of MCTD, which is more frequently seen. Patients with myositis associated with SS usually respond well to steroids and immunosuppressive therapy as reported in previous case reports [3, 9]. A similar response to immunosuppressive therapy was seen in our patient with a resolution of his myalgia and normalization of the CK. The patient is currently being followed up in the rheumatology clinic, receiving mycophenolate mofetil, along with IVIG (two-day course over a period of four months), methotrexate, prednisone, and folic acid, and remains asymptomatic .

## Conclusions

Herein, we describe a unique case of Sjögren's syndrome presenting with neuromuscular findings, including sensorimotor neuropathy. A subtle but definite muscle involvement was noted. Skeletal muscle involvement in primary SS is a relatively rare complication and the possibility of mixed connective tissue disease should be evaluated each time. A muscle biopsy may not be always fully contributory. This patient, however, responded favorably to high-dose steroids. Thus, we recommend considering (inflammatory) myopathy as part of the initial presentation of SS.
